# Rational Design of Safer Inorganic Nanoparticles via Mechanistic Modeling-informed Machine Learning

**DOI:** 10.21203/rs.3.rs-5960303/v1

**Published:** 2025-02-18

**Authors:** Joseph Cave, Anne Christiono, Carmine Schiavone, Henry J. Pownall, Vittorio Cristini, Daniela I. Staquicini, C. Jeffrey Brinker, Matthew J. Campen, Zhihui Wang, Hien Van Nguyen, Achraf Noureddine, Prashant Dogra

**Affiliations:** 1 Mathematics in Medicine Program, Department of Medicine, Houston Methodist Research Institute, Houston, TX, USA; 2 Physiology, Biophysics, and Systems Biology Program, Graduate School of Medical Sciences, Weill Cornell Medicine, New York, NY, USA; 3 Department of Electrical Engineering and Computer Science, Massachusetts Institute of Technology, Cambridge, MA, USA; 4 Department of Chemical, Materials, and Industrial Production Engineering, University of Naples Federico II, Naples, Italy; 5 Department of Medicine, Houston Methodist, Houston, TX, USA; 6 Department of Medicine, Weill Cornell Medicine, New York, NY, USA; 7 Neal Cancer Center, Houston Methodist Research Institute, Houston, TX, USA; 8 Department of Imaging Physics, University of Texas M.D. Anderson Cancer Center, Houston, TX, USA; 9 Rutgers Cancer Institute, Newark, NJ, USA; 10 Division of Cancer Biology, Department of Radiation Oncology, Rutgers New Jersey Medical School, Newark, NJ, USA; 11 Department of Chemical and Biological Engineering, University of New Mexico, Albuquerque, NM, USA; 12 College of Pharmacy, University of New Mexico, Albuquerque, NM, USA; 13 Department of Physiology and Biophysics, Weill Cornell Medical College, New York, NY, USA; 14 Department of Electrical and Computer Engineering, University of Houston, Houston, TX, USA

**Keywords:** nanoparticle, cytotoxicity, mathematical modeling, machine learning, artificial intelligence, PBPK, nanotoxicity

## Abstract

The safety of inorganic nanoparticles (NPs) remains a critical challenge for their clinical translation. To address this, we developed a machine-learning (ML) framework that predicts NP toxicity both *in vitro* and *in vivo*, leveraging physicochemical properties and experimental conditions. A curated *in vitro* cytotoxicity dataset was used to train and validate binary classification models, with top-performing models undergoing explainability analysis to identify key determinants of toxicity and establish structure-toxicity relationships. External testing with diverse mesoporous silica NPs validated the framework’s predictive accuracy for *in vitro* settings. To enable organ-specific toxicity predictions *in vivo*, we integrated a physiologically-based pharmacokinetic (PBPK) model into the ML pipeline to quantify NP exposure across organs. Retraining the ML models with PBPK-derived exposure metrics yielded robust predictions of organ-specific nanotoxicity, further validating the framework. This PBPK-informed ML approach can thus serve as a potential Novel Alternative Method (NAM) to streamline NP safety assessment, enabling the rational design of safer NPs and expediting their clinical translation.

Inorganic nanoparticles (NPs) have emerged as versatile platforms in biomedical applications, offering unparalleled tunability and precise control over physicochemical properties, including size, morphology, zeta (ζ) potential, surface coating, surface chemistry, and core composition^1^. This flexibility underpins the design of next-generation inorganic NPs with advanced drug delivery capabilities^2^, such as superior drug encapsulation^3^, enhanced colloidal stability^4^, targeted delivery^5^, controlled release^6^, and stimuli-responsive release^7^. Beyond drug delivery, inorganic NPs enable a diverse range of applications, including bioimaging^8–10^, antimicrobial therapy^11^, photodynamic therapy^12^, biosensing^13^, enzyme-mimicking catalysis^14^, and theranostics^15, 16^.

Despite these promising features, translating inorganic NP technologies to clinical practice remains a formidable challenge^17^. A critical hurdle is the control of nano-bio interactions^18^, such as protein corona formation, opsonization, and immune cell recognition, which often trigger rapid sequestration of NPs by the mononuclear phagocytic system (MPS), primarily the liver and spleen^19, 20^. This early clearance diminishes NP accumulation at target sites^21, 22^ while increasing the risk of off-target toxicity^23–25^. Additionally, the delayed degradation of inorganic NPs and the potential toxicity of their degradation products pose significant long-term safety concerns^26^. Nano-bio interactions, along with the resulting exposure and toxicity profiles, are heavily influenced by the physicochemical properties of NPs, offering opportunities for rational design to optimize *in vivo* disposition and safety. For instance, physicochemical attributes such as particle size, ζ-potential, surface coating, and core composition dictate circulation half-life^27^, biodistribution^28^, and toxicity^29, 30^. Typically, smaller NPs persist longer in circulation, exposing unintended organs like the kidneys, while larger particles are rapidly sequestered by the MPS^31^. Such off-target accumulations can induce localized reactive oxygen species (ROS) generation^32^, causing oxidative stress and disrupting many cellular processes through DNA damage^33^, membrane disruption^34^, and apoptosis^35^. Moreover, charged NPs can amplify interactions with immune cells, further exacerbating inflammation and safety concerns^36^.

Addressing these challenges requires predictive tools that quantitatively link NP properties to toxicity outcomes, thereby facilitating the rational design of safer NPs for clinical applications. Although traditional quantitative structure-activity relationship (QSAR) models have proven successful for small molecules, they face significant limitations when applied to nanomaterials due to their unique physicochemical properties and complex nano-bio interactions. Early attempts at applying QSAR to metallic and metal oxide NPs incorporated molecular-level descriptors such as electronegativity and oxidation state but largely overlooked size-dependent phenomena and surface effects critical at the nanoscale^37^. This mismatch underscores the urgent need for nanospecific approaches that accommodate the surface characteristics, size, and the dynamic interactions distinguishing NPs from small molecules.

Machine learning (ML) has emerged as a powerful alternative, leveraging nano-relevant descriptors to improve toxicity predictions. Studies have demonstrated its utility across various NP systems. For instance, CatBoost models identified concentration, hydrodynamic size, and exposure time as key predictors for silica NP cytotoxicity^38^, while decision tree classifiers emphasized material chemistry, ζ-potential, and NP size for broader NP types^39^. Random forest models have similarly revealed composition, ζ-potential, and exposure time as critical toxicity determinants^40^. These findings underscore the power of ML to integrate physicochemical and experimental parameters for robust *in vitro* predictions, however many studies remain limited to single endpoints or specific NP types.

Expanding on cytotoxicity-focused models, studies have employed ML frameworks to address more complex toxicity endpoints and systemic interactions. For example, ML models have been used to assess pulmonary immune responses and NP lung burden, identifying parameters such as surface area, dose, and size as significant predictors^41^. Tree-based models have highlighted core type, size, and surface coatings for human lung cytotoxicity^42^, while *in vitro* studies in cells have found cell line, dose, and tissue as major toxicity determinants^43^. These efforts demonstrate ML’s adaptability to diverse toxicity contexts, although gaps remain in integrating dynamic *in vivo* processes.

Emerging approaches incorporating omics data and advanced descriptors offer deeper insights into nano-bio interactions. For example, Bayesian networks with transcriptomics data have revealed disruptions in DNA damage and cell cycle regulation^44^, while studies on silver^45^ and zinc^46^ oxide NPs highlight exposure duration, ζ-potential, and surface coatings as critical toxicity drivers. ML models integrated to omics data have leveraged the use of nanoscale and molecular descriptors, including lysosomal dissociation degree, ζ-potential, and particle size to predict immune cell toxicity^47^. Advanced algorithms, such as neural networks, have further refined predictions, capturing intricate interactions between NP properties and biological systems^48^.

Despite these advances, current ML-based NP toxicity models are frequently constrained by limited data, narrow descriptor coverage, and a focus on specific NP types, reducing their applicability. Additionally, their inability to account for organ-specific *in vivo* exposure diminishes their translational relevance. To address these challenges, we assembled diverse descriptors spanning physicochemical properties, testing conditions, and biological contexts to predict cytotoxicity of inorganic NPs. To our knowledge, this study encompasses the largest curated database to date and introduces a novel binary classification framework integrating ML and physiologically-based pharmacokinetic (PBPK) modeling to predict nanotoxicity under both *in vitro* and *in vivo* conditions. Through explainability analysis and experimental testing on mesoporous silica NPs in primary and established cell lines, this framework uncovers hierarchies of toxicity determinants and quantitative structure-toxicity relationships. By experimentally verifying our predictions, we enhance model generalizability, bridging the gap between computational forecasts and real-world outcomes. This standardized Novel Alternative Method (NAM)^49^ offers a transformative approach to inorganic NP safety-by-design, accelerating clinical translation and regulatory approval of next-generation nanomaterials.

## ML workflow and data curation and for *in vitro* nanotoxicity predictions

**Figure 1.**
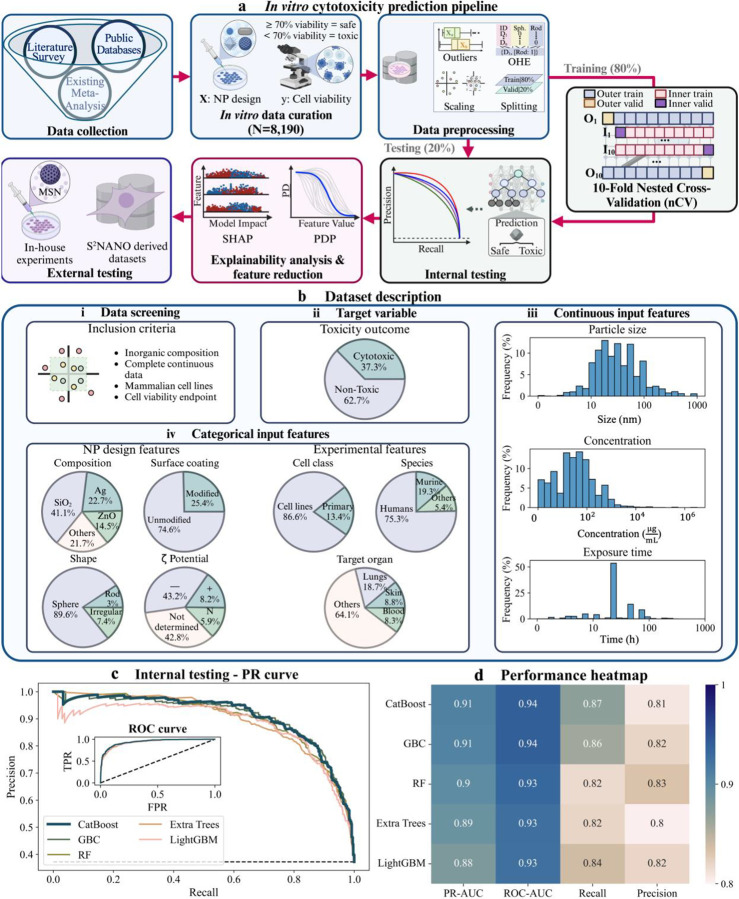
*In vitro* nanotoxicity prediction pipeline, dataset characterization, and machine learning (ML) model testing. **a)** The workflow for *in vitro* cytotoxicity predictions begins with data collection, resulting in a curated dataset of 8,190 samples. Data preprocessing includes harmonization of physicochemical descriptors, toxicity classification, scaling, and one-hot encoding (OHE) for ML model training and testing. The dataset is split into 80% training and 20% test subsets, with a nested cross-validation (nCV) framework applied to the training set. Internal testing is performed on the reserved test subset. Explainability analyses are employed to identify key toxicity drivers. External testing is performed using in-house experimental data based on mesoporous silica nanoparticles (MSNs) and additional data from the S^2^NANO repository. **b)** Dataset description and feature distributions. **(i)** Data inclusion criteria focus on studies reporting complete descriptors for inorganic NPs, including physicochemical properties, experimental conditions, and cell viability as a toxicity endpoint. **(ii)** Distribution of the target variable shows that 37.3% of samples were classified as cytotoxic, while 62.7% were non-toxic. **(iii)** Continuous input features include particle size, administered concentration, and exposure time, showcasing the wide variability in experimental conditions. **(iv)** Categorical input features include NP composition, surface coatings, ζ-potential, shape, cell class (primary or cell lines), and target organ. **c)** Internal testing results. Precision-recall (PR) curves demonstrate the performance of top ML models, including CatBoost, Gradient Boosting Classifier (GBC), Random Forest (RF), Extra Trees, and LightGBM. The inset receiver operating characteristic (ROC) curve shows true positive rates (TPR) versus false positive rates (FPR). Dashed black line in PR curve plot denotes the baseline precision for random guessing, while in ROC curve plot, it represents random classifier performance (FPR = TPR). **d)** Heatmap summarizing key testing metrics (PR-AUC, ROC-AUC, recall, and precision) for the best-performing models, highlighting the strong predictive capabilities of boosting and tree-based algorithms.

To address the need for robust predictive frameworks, we developed a computational workflow that integrates advanced ML tools for nanotoxicity prediction and curated a diverse dataset of inorganic NP cytotoxicity. The workflow outlined in Fig. 1a bridges data curation and computational modeling, starting with dataset harmonization and progressing through binary classification model training and testing. Explainability analyses were conducted to uncover key toxicity determinants, and external testing, leveraging in-house experimental data and the Safe and Sustainable Nanotechnology repository (S^2^NANO; www.s2nano.org), assessed model performance and generalizability. This integrative framework establishes a robust pipeline for predicting *in vitro* NP cytotoxicity, bridging computational predictions with experimental testing and advancing safety-by-design efforts for inorganic NPs.

The curated dataset comprises 8,190 samples extracted from 425 studies published between January 2004 and December 2023. These studies identified through systematic searches and prior meta- analyses^38, 39, 44–46, 50^, targeted *in vitro* toxicity of inorganic NPs in mammalian cell lines. Inclusion criteria focused on studies reporting essential descriptors, such as NP size, composition, concentration, surface coating, shape, ζ-potential, exposure time, and toxicity outcomes quantified via cell viability (Fig. 1b(i)).

The final dataset represents one of the most comprehensive collections for NP cytotoxicity studies, integrating diverse compositions, physicochemical properties, and experimental configurations (Fig. 1b(iii–iv)). Particle sizes spanned 1.5 to 1,000 nm, with SiO_2_ (41.1%), Ag (22.69%), and ZnO (14.51%) being the most common compositions. Surface coatings were predominantly unmodified (74.57%), while spherical geometries dominated (89.62%). ζ-potential, critical for assessing colloidal stability and biocompatibility, was reported as negative for 43.15% of NPs, neutral for 5.89%, and undetermined for 42.8%, reflecting data gaps. Experimental configurations varied widely, with exposure durations spanning 0.083 to 336 hours and administered concentrations from 5×10^−4^ to 3.2×10^6^ μg mL^−1^. Human-derived cells accounted for 75.27% of samples, with lungs (18.71%), skin (8.79%), and blood (8.33%) being the most studied organs, reflecting their relevance to primary exposure routes and systemic distribution. Harmonization aligned features across datasets, enabling consistent toxicity classification based on ISO 10993–5 standards (see Methods). Using the 70% viability threshold^38^, 62.74% of samples were classified as non-toxic and the remaining as toxic, indicating a moderate class imbalance (Fig. 1b(ii)).

## Boosting and tree-based models: optimal tools for nanotoxicity prediction

Building on the curated dataset’s diversity and comprehensive descriptor coverage, we systematically explored its potential for cytotoxicity classification using advanced ML approaches. A total of 18 algorithms were evaluated within a robust 10-fold nested cross-validation (nCV) framework (Fig. 1a), enabling precise hyperparameter tuning (**Table S1**) and effective overfitting control via early stopping where applicable (**Fig. S1**). Generalizability was assessed using a reserved 20% test subset, ensuring unbiased performance evaluation.

Given the class imbalance inherent in our dataset and the elevated cost of false negatives, we prioritized the precision-recall area under the curve (PR-AUC) and recall metrics over the receiver operating characteristic area under the curve (ROC-AUC) during evaluation. While ROC-AUC provides a general measure of classification performance, it often overestimates model capability in imbalanced scenarios^51, 52^. PR-AUC and recall better capture a model’s ability to identify toxic NPs while minimizing the risk of misclassification. Moreover, while PR-AUC is a comprehensive metric across thresholds, recall is particularly critical for high-stakes applications like nanotoxicity predictions, where minimizing false negatives is vital to avoid underestimating potential risks. This emphasis on recall shaped our model selection, as models with slightly lower PR-AUC but higher recall were prioritized to ensure sensitivity in detecting toxic NPs.

Among the tested algorithms, boosting and tree-based models emerged as the top performers, with superior PR-AUC values (0.8798–0.9098) and high recall rates (0.8164–0.8705) (Fig. 1c,d; **Fig. S2; Table S3**). The best performing models included CatBoost (PR-AUC: 0.9098, Recall: 0.8705), Gradient Boosting Classifier (GBC) (PR-AUC: 0.9079, Recall: 0.8607), Random Forest (RF) (PR-AUC: 0.8990, Recall: 0.8164), Extra Trees (PR-AUC: 0.8911, Recall: 0.8197), and LightGBM (PR-AUC: 0.8798, Recall: 0.8377) (Fig. 1d). Despite its marginally lower PR-AUC, LightGBM was favored over XGBoost due to its superior recall, highlighting its sensitivity in identifying toxic NPs (**Table S3**).

Conversely, models such as support vector machines (SVM), clustering algorithms, linear models, Naïve Bayes classifiers, and discriminant analysis exhibited poorer performance, characterized by higher false-negative and false-positive rates (**Figs. S2, S3; Table S3**). For example, SVC (PR-AUC: 0.8623, Recall: 0.7918), LDA (PR-AUC: 0.7403, Recall: 0.8246), and Logistic Regression (PR-AUC: 0.7501, Recall: 0.8410) showed limited reliability, underscoring their unsuitability for high-sensitivity applications such as nanotoxicity predictions.

To evaluate whether artificial neural networks (ANNs) offer advantages over traditional models, we conducted a comparative analysis using our top-performing boosting and tree-based algorithms as benchmarks. An ANN optimized via the Keras-Tuner framework achieved a PR-AUC of 0.9011, comparable to the best traditional models (**Fig. S4**; **Table S3**). However, its recall (0.7902) lagged behind, resulting in a higher misclassification rate for toxic NPs. While the ANN had predictive potential, its computational demands and reduced recall limit its practicality for preclinical applications. Boosting and tree-based models not only deliver superior sensitivity in detecting toxic NPs but also offer greater interpretability and computational efficiency, making them the preferred choice for scalable and explainable nanotoxicity predictions.

## SHAP analysis reveals concentration, composition, and size as key predictors of toxicity

Boosting and tree-based models not only deliver superior sensitivity in detecting toxic NPs but also offer greater interpretability and computational efficiency, making them the preferred choices for scalable and explainable nanotoxicity predictions. However, understanding why these models make specific predictions is crucial for translating computational insights into actionable guidelines for NP safety-by-design. To this end, we employed SHAP (SHapley Additive exPlanations)^53^, a widely adopted algorithm for feature attribution in ML, to quantify the relative importance of input features in predicting NP toxicity.

**Figure 2.**
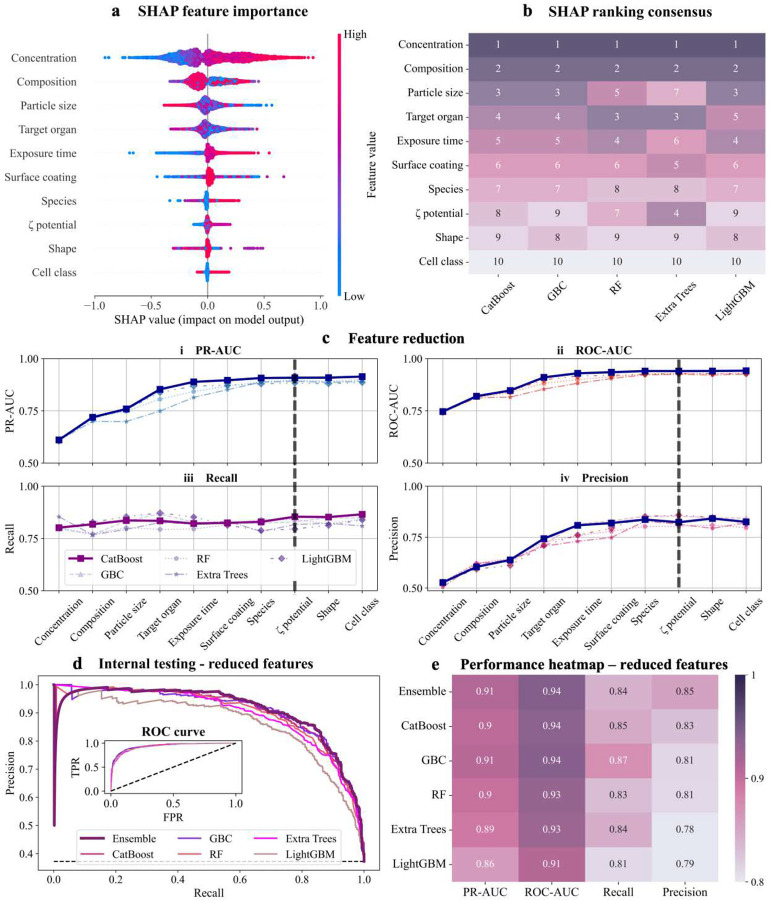
Explainability analysis, feature reduction, and internal testing of reduced-feature models. **a)** SHapley Additive exPlanations (SHAP) analysis for CatBoost, visualized as a beeswarm plot. Each point represents an individual prediction, highlighting the direction and magnitude of each feature’s contribution to NP toxicity classification. Higher SHAP values indicate greater importance, with features like concentration, composition, and particle size emerging as the most influential determinants of toxicity. **b)** SHAP consensus rankings across the top-performing models (CatBoost, GBC, RF, Extra Trees, LightGBM). The heatmap highlights high inter-model agreement, with concentration, composition, and particle size consistently ranked as the top three predictors. **c)** Iterative feature reduction results for CatBoost, visualizing changes in PR-AUC **(i)**, ROC-AUC **(ii)**, recall **(iii)**, and precision **(iv)** as features are added in descending order of SHAP importance. The solid black line denotes the point of performance saturation, beyond which adding additional features provides minimal improvement in predictive performance. **d)** Internal testing of top-performing models using the reduced feature set, evaluated through PR curves and ROC curves. The PR curves demonstrate strong predictive power with minimal loss compared to full-feature models, while the inset highlights ROC curves for these models. Dashed black line in PR curve plot denotes the baseline precision for random guessing, while in ROC curve plot, it represents random classifier performance (FPR = TPR). **e)** Performance heatmap summarizing internal testing metrics (PR-AUC, ROC-AUC, recall, precision) for top-performing models with reduced features.

Using CatBoost, our best-performing model, we analyzed the relative feature influence on NP toxicity predictions through SHAP values. Concentration in culture media, NP composition, and particle size emerged as the top three predictors of cytotoxicity (Fig. 2a). These features consistently ranked highest across the top-performing models, highlighting their critical role in determining NP toxicity (Fig. 2b). Secondary influential features, including target organ, exposure time, and surface coating also contributed to predictive performance but to a lesser degree compared to the top three factors (Fig. 2a, 2b).

In contrast, features such as animal species, ζ-potential, particle shape, and cell class had minimal influence, as reflected by low SHAP values (Fig. 2a, 2b). While ζ-potential is widely recognized for its role in influencing colloidal stability, cellular uptake, and protein corona formation, its low ranking in our analysis may stem from several factors. Approximately 43% of entries in our dataset lack defined ζ-potential values (Fig. 1b(iv)), limiting the model’s ability to extract meaningful patterns. Furthermore, ζ-potential alone may not fully capture the complexity of NP surface interactions, as suggested by prior studies highlighting surface charge density as a more reliable predictor of toxicity than ζ-potential alone^54^. The diversity of surface modifications across our dataset may have also diminished ζ-potential’s relative importance, as other surface properties could play a more prominent role in specific NP subgroups. Similarly, although particle geometry is often considered a key determinant of toxicity due to its effects on circulation half-life, endocytosis, immune response, and cell membrane disruption^55^, its reduced importance here can be attributed to the homogeneity of this feature in our dataset. With 89.62% of NPs being spherical (Fig. 1b(iv)), compounded by our assumption of spherical geometry when unspecified, this feature lacked sufficient variability to provide discriminative power. Lastly, cell class (i.e., primary *versus* cell lines) exhibited minimal influence, likely due to the limited variability in cell type composition within the dataset. With 87% of the entries corresponding to immortalized cell lines and only 13% to primary cells (Fig. 1b(iv)), the predictive utility of this feature in distinguishing toxicity outcomes was diminished.

To validate the reliability of the SHAP-derived feature hierarchy, we extended the analysis to the remaining top-performing models (Fig. 2b). The consensus of feature rankings was evaluated using the Spearman correlation coefficient, revealing strong alignment across models, with R>0.89. CatBoost exhibited excellent agreement with Gradient Boosting Classifier (R=0.9879), LightGBM (R=0.9758), and Random Forest (R=0.9515), supporting the robustness of the derived rankings. However, Extra Trees showed moderate agreement (R=0.7818) due to its greater emphasis on ζ-potential and lower prioritization of particle size. While this deviation highlights model-specific tendencies, the overall consistency reinforces the generalizability of the identified toxicity determinants.

This SHAP analysis not only elucidates the key drivers of NP toxicity but also provides actionable insights for the rational design of safer NPs. By focusing on key features like composition and particle size, which indirectly influence exposure levels and cellular interactions, researchers can optimize NP formulations to minimize cytotoxicity and enhance their translational potential. Furthermore, SHAP’s feature rankings present an opportunity to streamline predictive models by identifying and retaining only the most impactful features, reducing model complexity while maintaining accuracy.

## SHAP-guided feature reduction and model optimization

Building upon the SHAP analysis, we implemented an iterative retraining strategy to identify the minimal subset of features required for robust model performance. Features were progressively added to the top-performing models in descending order of SHAP importance, and changes in PR-AUC, ROC-AUC, recall, and precision were monitored at each step (Fig. 2c). This process revealed a performance plateau in PR-AUC, ROC-AUC, and precision after incorporating eight features: NP concentration, composition, particle size, target organ, exposure time, surface coating, species, and ζ-potential. These features proved essential for maintaining high predictive performance. In contrast, features such as shape and cell class demonstrated negligible impact, confirming their limited role in toxicity predictions.

Strategically reducing features enhances interpretability and computational efficiency while retaining predictive accuracy. The reduced-feature models achieved PR-AUC values above 0.8619 and recall exceeding 0.8098 across all top-performing algorithms (Fig. 2d, 2e; **Table S3**). The ROC curves further validate the minimal performance loss, showing near-identical trends between the full-feature and reduced-feature models (**inset** Fig. 2d). This consistency underscores the effectiveness of SHAP-guided feature reduction in simplifying models without compromising their predictive power.

The streamlined models offer significant advantages for preclinical deployment by reducing computational demands and enhancing interpretability. Additionally, the reduced model minimizes the number of features required from experimental measurements. By prioritizing eight key features, the model enables researchers to streamline experimental workflows, reducing the need to collect data on low-impact features. This not only enhances cost-efficiency but also accelerates NP screening and design, making the model highly suitable for preclinical and translational applications. These findings highlight the practical utility of explainable AI in toxicity modeling, providing a scalable framework for NP safety evaluation and rational design.

## Feature-specific insights into nanotoxicity: a framework for safety-by-design

**Figure 3.**
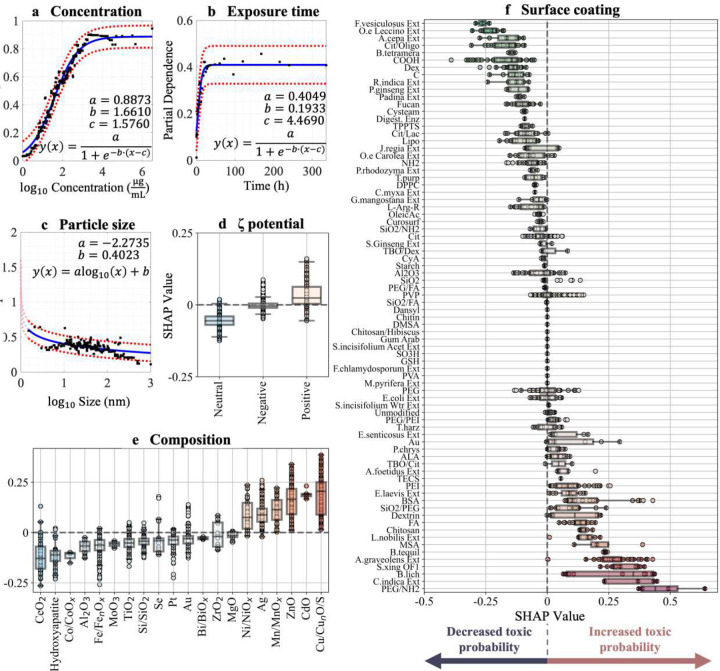
Feature-specific explainability analysis to inform NP safety-by-design strategies. **a-c)** Partial dependence plots (PDPs) depict the marginal effects of continuous features—NP concentration **(a)**, exposure time **(b)**, and particle size **(c)**—on predicted toxicity probabilities, holding all other features constant. Black dots represent data points, solid blue lines indicate model fits, and red dashed lines denote 95% confidence intervals. Empirical functions are provided to describe observed trends. **d-f)** SHAP summary plots illustrate the contribution of categorical features—ζ-potential **(d)**, NP composition **(e)**, and surface coating **(f)**—to toxicity predictions. Positive SHAP values indicate an increased probability of cytotoxicity, whereas negative values suggest reduced toxicity.

Building upon these insights, we leveraged the reduced features set to establish guidelines for safety-by-design. Using Partial Dependence Plots (PDPs) and SHAP, we systematically analyzed how variations in the top-ranked features influence NP toxicity. Concentration of NPs in culture media demonstrated a strong positive correlation with toxicity, showing a sigmoidal relationship as identified through regression analysis (Fig. 3a). The regression model indicates a rapid increase in toxicity probability at concentrations exceeding 10 μg mL^−1^, saturating at higher values. Similarly, exposure time followed a sigmoidal relationship, with a rapid increase in toxicity probability observed within the first 50 hours, followed by a gradual plateau around 100 hours (Fig. 3b). This trend suggests that toxic effects primarily manifest early during exposure, though some effects may persist over longer durations depending on experimental conditions.

Our findings also reveal that the probability of a toxic prediction decreases following a power-law decay with particle size, resulting in a linear trend when plotted against log10-transformed particle size (Fig. 3c). This trend aligns with existing evidence that smaller NPs are more toxic due to their higher surface area-to-volume ratio. For example, Song et al. demonstrated that smaller TiO_2_ NPs (~25 nm) induce significant ER stress and apoptosis in HepG2 cells, in contrast to larger NPs (~100 nm)^56^. Similarly, Pan et al. found that 1.4 nm AuNPs exhibit heightened genotoxicity across cell types by interacting with the major groove of B-DNA, disrupting transcription and initiating cell death within 12 hours^57^.

Further, positively charged NPs exhibited an increased propensity for toxic predictions compared to neutral or anionic counterparts, as shown by higher SHAP values for cationic particles (Fig. 3d). This effect can be attributed to stronger electrostatic interactions with negatively charged cell membranes, enhancing cellular uptake^54^ or destabilizing cell membranes^58^. Once internalized, these NPs dysregulate intracellular processes, particularly within the mitochondrial electron transport chain and endoplasmic reticulum (ER), leading to oxidative stress, cellular damage, and genotoxic effects, including prolonged arrest in the G0/G1 phase^59^. This ζ-potential-toxicity relationship aligns with literature^60^, including Hühn et al.’s findings that 3T3 fibroblasts rapidly internalized cationic AuNPs, resulting in elevated ROS levels^61^.

Additionally, NP composition was also a critical determinant of toxicity (Fig. 3e). Heavy metalbased NPs such as Cd, Cu, ZnO, Mn, Ni, and Ag exhibited higher SHAP values, indicating increased toxicity likelihood. In contrast, compositions like Ce, hydroxyapatite, Co, Al, and Fe were associated with greater safety. The release of toxic ions, modulated by microenvironmental factors like pH and ionic strength, underlies this trend. Toxic ions such as Ag^+^ cause direct cellular damage, while biologically essential ions like Fe^2+^ can be harmful at high concentrations^59^. These findings are consistent with Kobayashi et al., who evaluated the cytotoxic potential of 12 inorganic NPs and ranked Cd NPs as the most toxic^62^.

Surface coatings further modulate toxicity outcomes, with certain coatings reducing toxicity by preventing ion release or altering surface charge (Fig. 3f). For example, polyethylene glycol (PEG) coatings have been shown to improve colloidal stability and reduce cellular uptake, mitigating toxicity^63^. However, the large variability in SHAP values across surface coatings underscores the importance of considering interdependent factors, such as geometry and composition, which collectively influence properties like colloidal stability, ion release, and cellular interactions.

While PDP and SHAP analyses elucidate the marginal effects of individual features on toxicity predictions, NP toxicity is inherently multifactorial. The nonlinear interactions between physicochemical properties (e.g., size, charge, composition, coating) and testing conditions (e.g., concentration, exposure time) necessitate an ML model to accurately capture these dynamics. The integration of SHAP and PDP with ML enables both accurate predictions and interpretable insights, providing a robust framework for understanding and mitigating NP toxicity.

The large variability in SHAP values, particularly for NP coatings and composition, underscores the importance of considering the interdependence of physicochemical properties across heterogeneous experimental protocols^64^. For instance, surface coatings modify surface charge^65^, colloidal stability, and hydrodynamic size^59^, while geometry influences systemic half-life, organ bioaccumulation, and endocytosis mechanisms. Composition further affects surface chemistry and reactivity, including crystal structure, which can impact toxicity outcomes^66^.

Thus, our guidelines necessitate a holistic approach to safety-by-design, as previously demonstrated by Wu et al., who showed a size-dependent toxicity relationship in ultrasmall superparamagnetic iron oxide NPs (USPIONs)^67^. Their study showed USPIONs of 2.3 nm and 4.2 nm localized in lysosomes in cardiac tissue after IV administration in mice, releasing Fe^2+^ ions under acidic conditions. This release inversely correlated with NP size and catalyzed Fenton reactions, producing hydroxyl radicals (·OH) that led to oxidative stress and acute cardiac failure. In contrast, larger USPIONs (9.4 nm), as well as Au and SiO_2_ NPs, did not exhibit toxic effects. These findings highlight how size interacts with composition and the biological microenvironment to determine toxicity, underscoring the multifactorial nature of NP safety. This example demonstrates why a holistic framework, integrating key physicochemical properties and testing conditions, is critical for designing NPs that balance safety and functionality.

## External testing of predictive models for *in vitro* cytotoxicity

**Figure 4.**
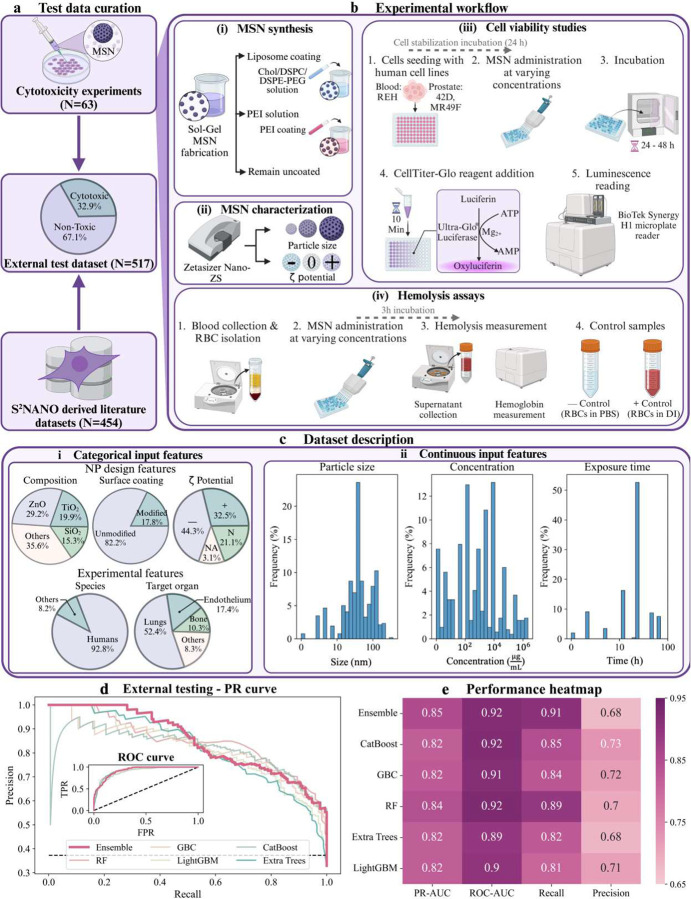
*In vitro* cytotoxicity data generation and external testing of ML model generalizability. **a)** Overview of test data sources, comprising in-house cytotoxicity experiments (N=63) and additional external testing data from the rigorously curated S^2^NANO repository (N=454), resulting in a combined external dataset (N=517) for testing. **b)** Experimental workflow for in-house cytotoxicity studies: **(i)** MSN synthesis using sol-gel fabrication and subsequent functionalization with lipid or polyethyleneimine (PEI) coatings; **(ii)** Characterization of MSNs by hydrodynamic size and ζ-potential measurements; **(iii)** Cell viability assays performed on human cell lines (REH, 42D, MR49F) using ATP-based luminescence readings following NP exposure; **(iv)** Hemolysis assays involving red blood cell (RBC) isolation and NP exposure, with phosphate buffer saline (PBS, negative control) and distilled water (DI water, positive control) validating assay accuracy. **c)** Dataset description: **(i)** Distribution of categorical input features, including NP composition, surface coating, ζ-potential, species, and target organ; **(ii)** Continuous feature distributions for particle size, concentration, and exposure time. **d)** External testing results presented as PR and ROC curves for the top-performing models (CatBoost, Gradient Boosting Classifier (GBC), Random Forest (RF), Extra Trees, LightGBM) and the ensemble model. The dashed black line in the PR curve plot denotes the baseline precision for random guessing, while in the ROC curve plot, it represents random classifier performance (FPR = TPR). **e)** Performance heatmap summarizing metrics, including PR-AUC, ROC-AUC, recall, and precision, highlighting the robust external testing and generalizability of the ensemble model, which achieved high recall and overall strong predictive performance.

Building on the feature-specific insights from the previous section, we evaluated the real-world utility of our ML framework in predicting NP toxicity. While the integration of SHAP and PDP analyses established a comprehensive safety-by-design framework, validating its generalizability across diverse datasets and experimental conditions remains essential. To address this, we performed external testing with in-house experimental data derived from cytotoxicity and hemolysis assays using mesoporous silica nanoparticles (MSNs). MSNs were specifically chosen due to their tunable physicochemical properties, which our team has extensive expertise in tailoring during synthesis^68–70^. This allowed us to systematically generate a diverse set of well-characterized MSNs, encompassing variations in size, porosity, and surface functionalities. These particles served as an ideal model nanomaterial for robust experimental testing. The experimental workflow is depicted in Fig. 4a, 4b, and detailed protocols are provided in **Supplementary Methods S1**.

To extend the scope of testing, we incorporated high-quality entries from the S^2^NANO repository. This rigorously curated database contains NPs of diverse compositions, exposure scenarios, and experimental conditions, enabling a broader assessment of the predictive capabilities of our framework. The merged dataset of 517 samples (63 in-house and 454 from S^2^NANO) reflects a balance between cytotoxic (32.9%) and non-toxic (67.1%) samples (Fig. 4a). This balance ensures that model performance is evaluated across both high- and low-risk samples, simulating realistic testing conditions. Importantly, the dataset encompasses diverse NP compositions (ZnO, SiO2, TiO2), surface coatings, and ζ-potential values, as well as a wide range of particle sizes, concentrations, and exposure times (Fig. 4c).

The external testing results, as shown in Fig. 4d and 4e, illustrate the predictive power of the ML models using both PR and ROC curves. The models yielded robust predictive performance on the external dataset, with PR-AUC values ranging from 0.82 to 0.85 and recall values from 0.81 to 0.91 (Fig. 4e, **Table S3**). Among the individual models, Random Forest (RF) emerged as the top performer, achieving a PR-AUC of 0.84 and a recall of 0.89, surpassing its internal testing metrics (recall: 0.84). In contrast, CatBoost, which excelled in internal testing, did not perform as well during external testing, achieving a PR-AUC of 0.82 and a recall of 0.85. These discrepancies highlight the importance of external testing in identifying models that generalize effectively beyond their training datasets.

ROC curves provided complementary insights into the models’ classification capabilities, balancing sensitivity (true positive rate) and specificity (false positive rate) (**inset** Fig. 4d). Across all models, ROC-AUC values remained consistently high, ranging from 0.89 to 0.9247. Among the individual models, RF also achieved the highest ROC-AUC of 0.9247, followed closely by CatBoost (ROC-AUC: 0.9214) and GBC (ROC-AUC: 0.9147). While LightGBM had slightly lower performance with an ROC-AUC of 0.9010, all models exceeded 0.89, confirming their reliability in external testing scenarios (Fig. 4e, **Table S3**).

To mitigate variability in individual model performance and reduce dependency on any single algorithm, we integrated the top-performing models into a unified stacking ensemble. By leveraging the complementary strengths of each base model, the ensemble achieved superior overall performance, with a PR-AUC of 0.85, a recall of 0.91, and a ROC-AUC of 0.92 (Fig. 4e, **Table S3**). While the ensemble underperformed in precision compared to CatBoost (0.73), GBC (0.72), RF (0.70), and LightGBM (0.71), it achieved the highest recall among all models. This makes the ensemble particularly well-suited for applications where minimizing false negatives is critical, such as nanotoxicity predictions. By optimizing recall while maintaining competitive overall performance, the ensemble model ensures robust generalizability and addresses the limitations of individual models, particularly in high-stakes preclinical testing scenarios.

The ensemble model’s robust performance across diverse experimental conditions and NP compositions signifies a pivotal advancement in *in vitro* nanotoxicity prediction. By replacing traditional trial-and-error approaches with a data-driven framework, our methodology supports the rational design of safer and more effective nanomedicines. These findings highlight the potential for deploying ML-driven strategies in nanomedicine, with far-reaching implications for both clinical translation and regulatory approval processes.

## Extending ML frameworks to predict *in vivo* nanotoxicity

**Figure 5.**
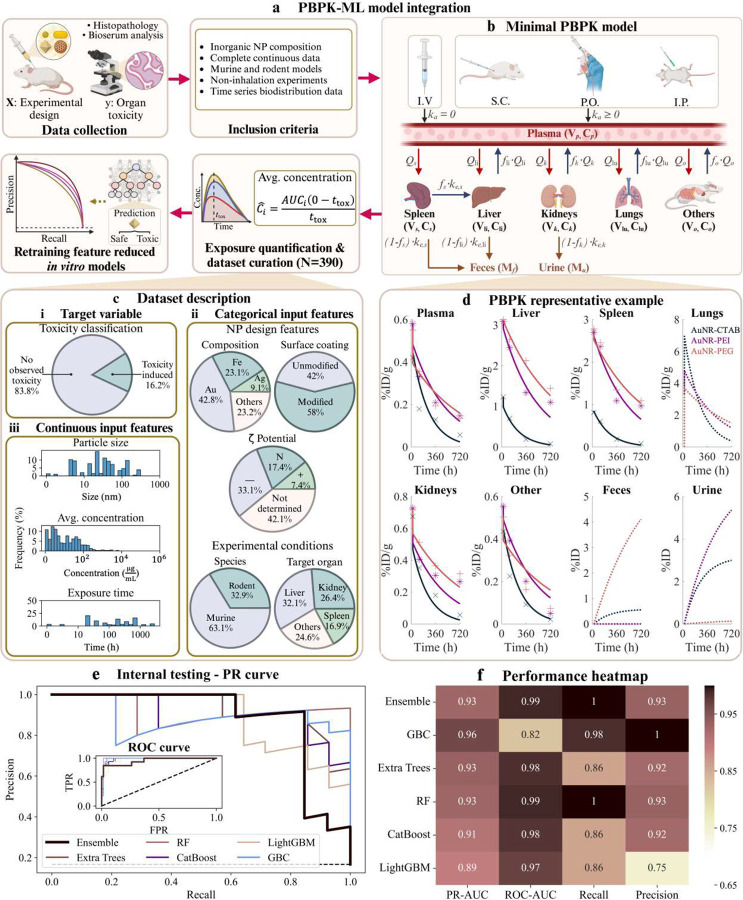
PBPK-ML framework for predicting in vivo nanotoxicity. **a)** Overview of the PBPK-ML model integration pipeline. Data curation involved selecting 390 samples based on inclusion criteria, including NP composition, murine/rodent models, and time-series biodistribution data. Time-averaged NP concentrations derived from the PBPK model were incorporated into retrained ML models previously optimized for *in vitro* predictions. **b)** Schematic of the minimal PBPK model, illustrating NP biodistribution across organs (plasma, spleen, liver, kidneys, lungs, and others) and clearance via feces and urine following intravenous (IV), subcutaneous (SC), oral (PO), or intraperitoneal (IP) administration. **c)** *In vivo* dataset description: **(i)** Toxicity outcomes, showing a majority (83.8%) with no observed toxicity; **(ii)** Categorical input features, including NP composition, surface coating, ζ-potential, species, and target organs; **(iii)** Continuous input features, such as particle size, concentration, and exposure time. **d)** Representative PBPK model concentration kinetics fits for gold nanorods (AuNR) with various surface coatings, showing excellent agreement with experimental data (Pearson correlation coefficients >0.98). **e)** Internal testing results for PBPK-ML models using PR and ROC curves, highlighting the performance of the top algorithms. Dashed black line in PR curve plot denotes the baseline precision for random guessing, while in ROC curve plot, it represents random classifier performance (FPR = TPR). **f)** Performance heatmap showing key metrics (PR-AUC, ROC-AUC, recall, and precision) for individual models and the ensemble model. The ensemble model achieved the highest accuracy, with PR-AUC = 0.93 and recall = 1.00, demonstrating the robustness of the PBPK-ML framework for organ-specific nanotoxicity predictions.

Leveraging the strength of our *in vitro* ML framework, we extended its applicability to *in vivo* settings by retraining it on curated *in vivo* nanotoxicity data. This adaptation bridges the gap between *in vitro* studies and complex *in vivo* dynamics, capturing the influence of organ-specific exposure and nano-bio interactions on toxicity outcomes. To achieve this, we incorporated a PBPK modeling approach^71–73^ to quantify time-averaged NP concentrations in individual organs. PBPK modeling incorporates the transport phenomena associated with NP biodistribution and simulates physiologically meaningful whole-body concentration-time profiles (Fig. 5b; **Equations S1–S8, Supplementary Methods S2**). This allowed us to capture the nuances of *in vivo* exposure across diverse experimental conditions and study designs. The resulting exposure metrics replaced the concentration feature used in the *in vitro* ML framework, enabling a seamless extension of the ML models to *in vivo* settings while maintaining its predictive robustness (Fig. 5a).

The minimal PBPK (mPBPK) model developed here, comprising six compartments (plasma, liver, spleen, lungs, kidneys, and others), successfully simulated NP biodistribution kinetics across diverse physicochemical and physiological conditions (Fig. 5c). Physiological parameters were either known *a priori*^74, 75^ (**Table S4**) or estimated through non-linear least squares fitting (**Table S5**), achieving Pearson correlation coefficients >0.9 for all simulations (Fig. 5d and **Fig. S5**). Time-averaged concentration of NPs across organs in the various studies estimated from the simulated concentration-time curves (${\hat{C}}_{l}=\frac{{\text{AUC}}_{i}\left(0-t_{\text{tox}}\right)}{t_{\text{tox}}}$; see Methods) ranged from 0.01 to 7185.65 μg mL^−1^ (Fig. 5c(iii)), capturing diverse exposure scenarios relevant to both acute and chronic toxicity assessments. This metric consolidates temporal dynamics into a single representative value, enabling direct comparisons across studies with varying exposure durations. The mPBPK model offers a computationally efficient approach to quantify NP biodistribution, guiding preclinical NP design for enhanced safety.

Using a curated dataset of 390 samples derived from 35 studies (Fig. 5c), detailed NP biodistribution data enabled the parameterization of the mPBPK model and the retraining of the ML framework. This dataset encompassed diverse physicochemical properties, including gold (42.8%), iron oxide (23.1%), and silver (9.1%) NPs, with a range of surface modifications and organ-specific exposure patterns. The integration of PBPK-derived metrics enhanced the framework’s ability to predict organ-specific *in vivo* toxicity, as demonstrated by robust model performance (Fig. 5e, 5f).

Individual models were characterized by strong testing performance, with PR-AUC values ranging from 0.89 to 0.96 and recall values from 0.86 to 1.0 (Fig. 5f, **Table S3**). Among these, GBC achieved the highest PR-AUC (0.96) with a recall of 0.98, while RF attained perfect recall (1.0) but a slightly lower PR-AUC (0.93) due to reduced precision. Extra Trees balanced precision and recall effectively, with a PR-AUC of 0.93 and a recall of 0.86. The stacking ensemble strategy leveraged the strengths of individual models, achieving a robust PR-AUC of 0.93 and perfect recall (1.0), demonstrating its ability to enhance generalizability and mitigate biases in individual models.

Complementary ROC analysis (**inset** Fig. 5e) confirmed the high predictive reliability of the models. The stacking ensemble achieved an outstanding ROC-AUC of 0.99, indicating near-optimal classification performance across all false positive rates. Similarly, RF matched the ensemble’s performance with an ROC-AUC of 0.99, while Extra Trees followed closely at 0.98. Both CatBoost and LightGBM demonstrated strong classification capabilities, achieving ROC-AUCs of 0.98 and 0.97, respectively. In contrast, GBC exhibited comparatively lower performance, with an ROC-AUC of 0.82, reflecting variability in its reliability. Collectively, these results underscore the robustness of the ensemble approach, effectively combining the strengths of individual models for highly accurate and generalizable nanotoxicity predictions.

The stacking ensemble’s consistent performance across PR-AUC and ROC-AUC metrics underscores its suitability for *in vivo* toxicity predictions. By mitigating individual model biases, the ensemble enhances reliability and generalizability across diverse datasets. These findings emphasize the PBPK-ML framework’s potential to bridge *in vitro* and *in vivo* toxicity assessments, facilitating the clinical adoption of safer, design-optimized NPs while minimizing toxicity risks.

## Conclusion

The clinical translation of inorganic NPs has been stymied by a lack of standardized frameworks to predict toxicity across diverse experimental settings. Conventional trial-and-error approaches, compounded by inconsistencies in data reporting, have limited progress in the design and deployment of safe and effective NPs. Addressing these challenges, we present a novel ML framework, enhanced by PBPK modeling, to predict NP toxicity both *in vitro* and *in vivo* with unprecedented precision and scalability.

Our ML framework, trained on the largest curated *in vitro* cytotoxicity dataset to date, achieved robust predictive performance, with ensemble models demonstrating PR-AUCs exceeding 0.89 and recalls above 0.9. Explainability analyses revealed NP concentration as the dominant predictor of toxicity, while other physicochemical features such as composition and size provided critical design insights. These findings informed safety-by-design principles, providing a quantitative foundation for rational NP development.

The integration of a PBPK model represents a significant step forward, enabling mechanistic insights into organ-specific NP biodistribution and its impact on toxicity. This mechanistic layer allowed for the generation of time-averaged exposure metrics, bridging *in vitro* predictions with the complexity of *in vivo* environments. Ensemble models retrained on curated *in vivo* datasets achieved outstanding predictive accuracy, with PR-AUCs and ROC-AUCs approaching optimal performance. This integration of mechanistic modeling and ML provides a scalable and physiologically meaningful approach to preclinical safety assessment.

While these advances represent a significant milestone, several limitations warrant consideration. The relatively small size of the *in vivo* dataset and variability in biodistribution reporting across studies posed challenges to the PBPK model’s generalizability. The exclusive focus on inorganic NPs limits the framework’s applicability to organic or hybrid nanomaterials, while the minimal PBPK model does not yet account for more complex nano-bio interactions, such as immune responses or protein corona formation. These constraints underscore the need for further data curation and mechanistic model refinements to enhance the framework’s scope and accuracy.

Looking ahead, this PBPK-informed ML framework offers a potential Novel Alternative Method (NAM) for preclinical safety assessments, providing a harmonized and scalable approach for evaluating NP toxicity. Future work will focus on addressing data gaps, particularly in biodistribution reporting, and expanding the framework to include organic NPs and hybrid materials. By bridging computational insights with experimental testing, this framework establishes a blueprint for rational NP design, accelerating the development of safer and more effective nanomedicines.

## Methods

### Data collection, curation, and preprocessing for *in vitro* cytotoxicity predictions

Our *in vitro* cytotoxicity dataset was assembled from previously published meta-analyses^38, 39, 44–46, 50^ and a systematic review of peer-reviewed articles (January 2004–December 2023) identified through Google Scholar, PubMed, and Web of Science. We applied strict inclusion criteria: (i) only inorganic NPs were considered; (ii) studies must provide NP size, exposure duration, and NP concentration data; (iii) the focus was on biomedical rather than environmental safety applications; (iv) experiments had to be conducted *in vitro* using mammalian cell lines; and (v) cytotoxicity had to be quantified via percentage cell viability. We extracted viability data using WebPlotDigitizer (https://automeris.io/). This screening yielded 425 papers (Fig. 1a).

To ensure data harmonization, particle sizes were standardized to nanometers (nm), exposure durations to hours (h), and concentrations to micrograms per milliliter (μg mL^−1^). For missing categorical variables, surface coating was labeled “Unmodified” and shape was labeled “Sphere” where not reported. ζ-potential was categorized as positive (ζ > 10 mV), negative (ζ < −10 mV), neutral (−10 mV ≤ ζ ≤ 10 mV), or “not determined,” prioritizing measurements in deionized water (or culture media if water measurements were unavailable).

Following ISO 10993–5 guidelines, the cell viability endpoint was binarized such that ≥70% viability was labeled “safe” (assigned 0), and lower viability was labeled “cytotoxic” (assigned 1)^38^. This encoding was performed using Pandas (Python). Next, each continuous variable (particle size, concentration, and exposure time) underwent outlier detection using a 1.5× interquartile range cutoff; flagged outliers were manually inspected and removed if deemed unreliable or nonsensical.

The resulting dataset was then shuffled to eliminate order bias and split into training (80%) and test (20%) subsets via train_test_split() in Scikit-Learn (v1.5.1). We applied a logarithmic transformation to each continuous variable to mitigate skewness and stabilize variance. Categorical variables underwent one-hot encoding using Scikit-Learn, omitting the least frequent category to avoid collinearity (i.e., the “dummy variable trap”; **Table S2**). Finally, to handle the expanded feature space after one-hot encoding, we converted the DataFrame into a sparse matrix using SciPy(v1.13.1), enabling efficient model training.

### Machine learning (ML) pipeline for *in vitro* cytotoxicity predictions

#### Model selection and implementation

A diverse array of 18 binary classification algorithms was employed to predict the cytotoxicity of inorganic NPs. These models were implemented using the SciKit-Learn, CatBoost (v1.2.2), LightGBM (v4.4.0), and XGBoost (v2.0.3) libraries in Python 3.12. To ensure reproducibility, a random state of 3 was set. The suite of algorithms spanned multiple methodological classes, including boosting and tree-based models (such as AdaBoost, Gradient Boosting Classifier, and Random Forest), clustering algorithms (k-nearest neighbor and radius-neighbor classifiers), discriminant and kernel-based approaches (linear and quadratic discriminant analysis, support vector classifiers), linear models (logistic regression, perceptron, and stochastic gradient descent), and Naïve-Bayes classifiers. This comprehensive selection allowed for an unbiased evaluation of algorithmic efficacy, ensuring robustness across diverse methodologies.

#### Training and internal testing

Model training was performed on a Lenovo ThinkStation P520 equipped with an Intel Xeon W-2125 CPU and NVIDIA Quadro P4000 GPU, as well as a 2022 Apple MacBook Pro featuring an M2 chip. A 10-fold nested cross-validation (nCV) technique was implemented to ensure robust model evaluation and hyperparameter optimization. In the nCV framework, the outer loop assessed model performance, while the inner loop optimized hyperparameters using the StratifiedKFold, cross_validate, and GridSearchCV functions from the SciKit-Learn library.

The dataset was stratified into ten folds to maintain a consistent class distribution across folds. During each iteration of the outer loop, nine folds were used for training and one for testing. Hyperparameters were optimized in the inner loop to maximize the area under the precision-recall curve (PR-AUC), a metric well-suited for imbalanced datasets. The list of hyperparameters optimized through grid search is provided in **Table S1**. Optimal hyperparameters identified from each fold of the inner loop were consolidated into a refined parameter grid, which was subsequently applied to retrain the models on the entire training data partition. This approach enhanced the robustness and generalizability of the final models by focusing on the most effective parameter combinations.

Early stopping was incorporated to prevent overfitting for boosting models (CatBoost, Gradient Boosting Classifier, LightGBM, and XGBoost) that support history monitoring. Log-loss was monitored on a 10% hold-out subset of the training data, with early stopping triggered after 50 consecutive iterations without improvement, up to a maximum of 5,000 boosting rounds. For models not employing early stopping, default settings for the number of estimators were used.

To further evaluate model performance, an unseen 20% of the dataset was reserved as an internal test set. The decision threshold was programmatically adjusted using threshold tuning to maximize the F1 score, balancing precision and recall for imbalanced datasets. Evaluation metrics, calculated using the metrics package from SciKit-Learn, included accuracy $\left(\frac{\text{TP}+\text{TN}}{\text{TP}+\text{TN}+\text{FP}+\text{FN}}\right)$, ROC-AUC, sensitivity $\left(\frac{\text{TP}}{\text{TP}+\text{FN}}\right)$, specificity $\left(\frac{\text{TN}}{\text{TN}+\text{FP}}\right)$, F-1 score $\left(2\times \frac{\text{Precision}\times \text{Recall}}{\text{Precision}+\text{Recall}}\right)$, PR-AUC, Matthews Correlation Coefficient (MCC) $\left(\frac{\left(\text{TP}\times \text{TN})-(\text{FP}\times \text{FN}\right)}{\sqrt{\left(\text{TP}+\text{FP})\text{x}(\text{TP}+\text{FN})\text{x}(\text{TN}+\text{FP})\text{x}(\text{TN}+\text{FN}\right)}}\right)$, and Balanced Accuracy (BA) score $\left(\frac{\text{sensitivity}+\text{specificity}}{2}\right)$.

For the purpose of NP toxicity predictions, true positives (TP) referred to correctly identified toxic NPs, false positives (FP) to non-toxic NPs misclassified as toxic, true negatives (TN) to correctly identified non-toxic NPs, and false negatives (FN) to toxic NPs misclassified as non-toxic. Precision, defined as TP / (TP + FP), measured the proportion of true toxic predictions among all toxic predictions made, while recall (sensitivity), defined as TP / (TP + FN), quantified the proportion of correctly identified toxic NPs among all actual toxic NPs.

Performance metrics such as PR and ROC curves were visualized using the plot() function in Matplotlib (v3.9.1). Given the dataset’s imbalance and the high cost associated with false negatives, PR-AUC and recall were prioritized during model evaluation. The five best-performing models were selected based on their robustness in managing class disparity and their effectiveness in minimizing false negatives.

#### Artificial neural network

Neural network models were developed using the Keras-Tuner toolkit (v1.4.7; https://github.com/keras-team/keras-tuner) and TensorFlow (v2.10), with TensorFlow Metal employed for enhanced compatibility with Apple hardware. To ensure consistency across experiments, we implemented a 10-fold nested cross-validation (nCV) framework, mirroring the protocol used for traditional ML models. Four critical hyperparameters—learning rate, number of hidden layers, dropout rate, and type of regularization (L1, L2, or elastic net)—were optimized using Keras-Tuner’s GridSearch.

The model architecture followed a sequential design, beginning with an input dense layer containing a number of nodes equal to the dataset’s feature count (**Fig. S4**). Hidden layers, configured based on grid search results, consisted of 128 units each, with ReLU activation, batch normalization, and dropout layers applied consistently. Regularization was implemented using L1, L2, or elastic net techniques at a fixed strength of 0.01, enhancing the network’s ability to generalize across diverse data. The output layer utilized a sigmoid activation function to generate binary predictions, and the Adam optimizer was selected for its effectiveness in handling sparse gradients and noisy datasets. Binary_crossentropy was employed as the loss function, with PR-AUC as the primary evaluation metric to address the dataset’s class imbalance.

Training included a 10% validation split to monitor binary cross entropy loss, analogous to log-loss monitoring in traditional ML models. This ensured early detection of overfitting while maximizing the use of training data. After identifying optimal hyperparameters for each inner fold during nCV, the model was retrained on the full outer training fold before final evaluation on the held-out outer test fold. This systematic optimization and evaluation protocol ensured a thorough assessment of ANN performance, with results directly comparable to those from traditional ML models.

#### Model unification

To improve the accuracy and robustness of cytotoxicity predictions, we developed a unified model using a stacked ensemble classifier approach implemented with the SciKit-Learn library. This methodology integrates predictions from the five best-performing base models, which were selected based on their PR-AUC and recall performance during cross-validation. A logistic regression meta-model was employed as the stacking layer, enabling an equitable combination of base model outputs while leveraging their diverse predictive strengths. The choice of logistic regression as the meta-learner was motivated by its simplicity and effectiveness in managing multi-model integration without overfitting. Logistic regression operates on the probability outputs of the base models, ensuring smooth integration while maintaining interpretability. This design minimizes bias by distributing reliance across multiple models, improving generalizability and reducing the risk of overfitting that could arise from dependence on a single predictive model. By combining the unique strengths of individual classifiers, the stacking strategy enhances overall predictive accuracy and addresses the challenges of imbalanced data in NP cytotoxicity predictions. This unified approach ensures that the complementary insights of diverse models are systematically captured, offering a reliable and scalable solution for toxicity assessment.

### Explainability analysis

#### Global SHAP ranking

We performed explainability analysis using SHapley Additive exPlanations (SHAP, v0.44)^53^ to establish a global hierarchy of toxicity determinants. SHAP values quantify the contribution of individual features to model predictions, providing insights into their influence on toxicity outcomes. The SHAP value (*ϕ*_*i*_(*f*)) for a feature *i* is calculated as:

$$\phi_{i}(f)=\sum_{S\subseteq N}\frac{|S|!(|N|-|S|-1)!}{|N|!}\cdot [f(S\cup \{i\})-f(S)]$$

where *f* is the binary classifier model, *S* is a subset of all features excluding *i*, and *N* is the full feature set. This equation measures the average impact of including feature *i* across all feature subsets. SHAP values were computed using the TreeExplainer(output_argument=‘probability’) function, ensuring interpretations corresponded directly to the probabilistic model outputs.

To simplify interpretation, SHAP values for one-hot encoded categories were aggregated to calculate a single influence score for each feature. This aggregation allowed for direct featurelevel comparisons and enhanced interpretability. Beeswarm plots were generated using the summary_plot() function to visualize the distribution of SHAP values, with color encoding to represent the magnitude of feature influence. One-hot encoded features were preprocessed using the encoder function from SciKit-Learn to facilitate visualization. To assess the consensus of feature rankings across the top five models, we calculated Spearman correlation coefficients using the corr(method=‘spearman’) function from Pandas (v1.1.5). These rankings were visualized as a heatmap generated with the heatmap() function in Seaborn (v0.13.2).

#### Quantifying structure-toxicity relationships

We employed partial dependence plots (PDPs) to evaluate the marginal effects of key continuous variables, i.e., concentration, exposure time, and particle size, on predicted NP toxicity probabilities. PDPs were generated using the partial_dependence() function from SciKit-Learn and extended using regression analysis in MATLAB (v2023b) to fit empirical mathematical functions. Confidence intervals (95%) were calculated to quantify variability. For categorical features, SHAP values were computed separately for each category and filtered to isolate their specific contributions to predicting toxicity. Non-normal SHAP value distributions were summarized using the median, with positive median SHAP values indicating stronger associations with toxicity predictions and negative values reflecting decreased toxicity likelihoods. These distributions were visualized using boxplots created with Matplotlib and Seaborn.

### Feature reduction

To streamline our best-performing models and improve computational efficiency, we applied an iterative retraining strategy based on feature rankings derived from SHAP analysis. SHAP ranked features by their contribution to toxicity predictions but did not provide a specific cutoff to distinguish crucial features from non-essential ones. Therefore, we progressively incorporated features into the model in descending order of SHAP importance, retraining the model at each step and monitoring changes in performance metrics, specifically PR-AUC and recall. This iterative approach identified a performance plateau, beyond which the inclusion of additional features did not improve PR-AUC or recall, indicating their marginal contribution to toxicity predictions. The results from each iteration were visualized using Matplotlib, highlighting the relationship between feature inclusion and model performance.

Once the minimal subset of features was identified, final retraining phases were conducted on the best-performing models identified earlier (CatBoost, GBC, RF, Extra Trees, and LightGBM), using only the features deemed crucial. These reduced-feature models were trained and tested using the same nCV and internal testing processes as described previously to ensure consistency and reliability. Performance comparisons between full-feature and reduced-feature models were assessed to validate the efficacy of the reduction process.

### External testing for *in vitro* cytotoxicity predictions

To evaluate the generalizability and robustness of our ML models, we conducted external testing using two complementary sources of *in vitro* cytotoxicity data: (1) independent cell viability and hemolysis experiments conducted in-house and (2) supplemental entries from the S^2^NANO database, a peer-reviewed repository of extensively characterized NPs. Together, these datasets mimic real-world testing conditions and provide a comprehensive framework for validating toxicity predictions. Mesoporous silica nanoparticles (MSNs) were selected as the model nanomaterial for in-house studies, leveraging our team’s expertise in synthesizing MSNs with diverse physicochemical properties, including hexagonal and dendritic architectures with tailored size, porosity, and surface functionality^68^. Lipid and polyethyleneimine (PEI) coatings were applied to modulate surface charge and biocompatibility. Detailed descriptions of MSN synthesis, functionalization, characterization, and cytotoxicity assay protocols are provided in the **Supplementary Methods S1**.

To ensure broader applicability of our framework, we expanded external testing by incorporating entries from the S^2^NANO (www.s2nano.org) database, a rigorously curated resource containing NPs across diverse compositions and experimental contexts. The high P-scores assigned to database entries reflect their reliability and quality, sourced through established meta-analyses. This inclusion enabled testing of model performance on a wide array of NP types, including materials beyond MSNs and across various organ-specific cell lines. All entries were processed using the standardized preprocessing pipeline described earlier, ensuring consistency in feature extraction and toxicity endpoint definitions. Metrics such as PR-AUC, recall, and F1-score confirmed the robustness and predictive accuracy of the ML models across the combined dataset, reinforcing their utility for real-world applications.

### Developing an *in vivo* nanotoxicity prediction framework

To extend the predictive capabilities of our ML framework to *in vivo* settings, we integrated curated nanotoxicity data with a minimal physiologically based pharmacokinetic (mPBPK) model. This hybrid framework quantifies organ-specific NP exposure, enabling accurate toxicity predictions while accounting for both physiological and physicochemical factors. The approach involves comprehensive data curation, PBPK-based exposure quantification, and retraining the ML framework to enhance its generalizability across diverse preclinical scenarios. Organ-specific exposure metrics, derived from area under the curve (AUC) of PBPK simulations, replace the concentration feature used *in vitro*, bridging *in vitro* and *in vivo* predictions.

#### In vivo data curation

To develop robust and generalizable ML models for *in vivo* toxicity, we curated a dataset integrating toxicity and biodistribution data from studies published between January 2004 and May 2024. Data were sourced from Google Scholar, PubMed, and Web of Science. Studies were selected based on stringent inclusion criteria: they utilized murine and rodent models, focused on inorganic NPs with well-defined physicochemical properties (e.g., size, dose, and exposure duration), and employed non-inhalation administration routes (intravenous (IV), subcutaneous (SC), intraperitoneal (IP), and oral (PO)). Toxicity assessments included biochemical, hematological, or histopathological analyses compared to controls. Only studies reporting NP concentrations in organs at a minimum of three distinct time points were included, enabling accurate PBPK modeling. This rigorous selection process resulted in 35 high-quality studies, forming the foundation for PBPK model development and ML retraining (Fig. 5a).

The final curated dataset consisted of 390 samples, encompassing diverse physicochemical properties and biodistribution patterns. Predominant NP types included gold (42.8%), iron oxide (23.1%), and silver (9.1%), with particle sizes ranging from 1.2 to 310 nm. Surface modifications varied, with polyethylene glycol (PEG) coatings (27.44%) and unmodified NPs (42.05%) representing the majority. IV administration accounted for 90.77% of samples, and key organs such as the liver (32.05%), kidneys (26.41%), and spleen (16.92%) were most frequently analyzed due to their roles in NP metabolism and clearance.

#### PBPK model development

To simulate NP biodistribution and clearance dynamics *in vivo*, we developed a minimal PBPK model comprising six compartments: plasma, liver, spleen, lungs, kidneys, and others (Fig. 5b). The model accounted for perfusion-limited transport and first-order excretion kinetics, effectively capturing critical pharmacokinetics and transport phenomena (**Equations S1–S8**). Time-averaged NP concentrations for individual organs were derived from simulated concentration-time profiles and subsequently used as exposure metrics for ML training. Details on model equations, parameterization, and numerical implementation are provided in the **Supplementary Methods S2**.

#### Quantifying organ-specific NP biodistribution

Following the parameterization of the PBPK model, the time-averaged concentration $\left({\hat{C}}_{l}\right)$ was quantified for each compartment (*i*) using the equation:

$${\hat{C}}_{l}=\frac{{\text{AUC}}_{i}\left(0-t_{\text{tox}}\right)}{t_{\text{tox}}}$$

Here, AUC_*i*_(0–*t*_tox_) represents the area under the PBPK model’s concentration-time curve for compartment *i* up to the time of toxicity measurement (*t*_tox_). This time-averaged concentration metric provides a robust summary of NP biodistribution dynamics during the period relevant to toxicity assessments. This approach is particularly advantageous when comparing studies with varying experimental designs or exposure durations, as it consolidates temporal dynamics into a single representative value. To ensure the reliability of the simulated biodistribution metrics, only model simulations achieving a Pearson correlation coefficient R>0.9 between fitted and observed biodistribution data were included in the final dataset. This threshold criterion ensured the inclusion of only high-confidence simulations for subsequent *in vivo* toxicity prediction modeling.

#### ML framework retraining

To extend the ML framework from *in vitro* to *in vivo* settings, the curated dataset was split into 80% for training and 20% for testing. The same ML pipeline used for *in vitro* data was applied, employing nCV within the training set to optimize hyperparameters and ensure robust model performance. Specifically, a 10-fold nCV approach was implemented to optimize hyperparameters and assess generalization performance. The outer loop evaluated overall model generalization, while the inner loop fine-tuned hyperparameters for optimal performance. Only the top five performing models from the *in vitro* analysis, along with the stacking ensemble, were retrained to focus on models with demonstrated reliability and predictive power. Additionally, the integration of PBPK-derived time-averaged concentration metrics into the ML framework provided a physiologically meaningful representation of organ-specific NP exposure. This adaptation bridged the gap between *in vitro* and *in vivo* predictions, preserving methodological consistency while accommodating the complexities of *in vivo* dynamics. By concentrating on the most effective models and leveraging PBPK insights, the framework ensured robust and scalable predictions across biological contexts.
